# OIDA: An optimal interval detection algorithm for automatized determination of stimulation patterns for FES-Cycling in individuals with SCI

**DOI:** 10.1186/s12984-022-01018-2

**Published:** 2022-04-14

**Authors:** Martin Schmoll, Ronan Le Guillou, Charles Fattal, Christine Azevedo Coste

**Affiliations:** 1 INRIA-Université de Montpellier, Montpellier, France; 2 Rehabilitation Center Bouffard Vercelli, USSAP Perpignan, Perpignan, France

**Keywords:** Functional electrical stimulation (FES), FES-Cycling, Cybathlon, Spinal cord injury, FES-Sports, Inertial measurement unit (IMU), Muscle torque production

## Abstract

**Background:**

FES-Cycling is an exciting recreational activity, which allows certain individuals after spinal cord injury or stroke to exercise their paralyzed muscles. The key for a successful application is to activate the right muscles at the right time.

**Methods:**

While a stimulation pattern is usually determined empirically, we propose an approach using the torque feedback provided by a commercially available crank power-meter installed on a standard trike modified for FES-Cycling. By analysing the difference between active (with stimulation) and passive (without stimulation) torques along a full pedalling cycle, it is possible to differentiate between contributing and resisting phases for a particular muscle group. In this article we present an algorithm for the detection of optimal stimulation intervals and demonstrate its functionality, bilaterally for the quadriceps and hamstring muscles, in one subject with complete SCI on a home trainer. Stimulation patterns were automatically determined for two sensor input modalities: the crank-angle and a normalized thigh-angle (i.e. cycling phase, measured via inertial measurement units). In contrast to previous studies detecting automatic stimulation intervals on motorised ergo-cycles, our approach does not rely on a constant angular velocity provided by a motor, thus being applicable to the domain of mobile FES-Cycling.

**Results:**

The algorithm was successfully able to identify stimulation intervals, individually for the subject’s left and right quadriceps and hamstring muscles. Smooth cycling was achieved without further adaptation, for both input signals (i.e. crank-angle and normalized thigh-angle).

**Conclusion:**

The automatic determination of stimulation patterns, on basis of the positive net-torque generated during electrical stimulation, can help to reduce the duration of the initial fitting phase and to improve the quality of pedalling during a FES-Cycling session. In contrast to previous works, the presented algorithm does not rely on a constant angular velocity and thus can be effectively implemented into mobile FES-Cycling systems. As each muscle or muscle group is assessed individually, our algorithm can be used to evaluate the efficiency of novel electrode configurations and thus could promote increased performances during FES-Cycling.

**Supplementary Information:**

The online version contains supplementary material available at 10.1186/s12984-022-01018-2.

## Background

Cycling is a very attractive sportive discipline which unfortunately is not available for everyone. Due to various reasons (e.g. traumatic injuries, diseases, congenital…), certain individuals end up losing the voluntary control of their legs. In particular cases, functional electrical stimulation (FES) can be used to activate the paralyzed muscles by stimulating the remaining motor-nerves or the muscle itself [1].

Besides classical resistance training [2, 3], FES can also be used to allow individuals with a spinal cord injury (SCI) to access recreational activities such as cycling [4, 5]. In the meanwhile, FES-Cycling was established as an independent discipline during international competitions, such as at the Cybathlon [5, 6]. Crucial for this type of application is a coordinated temporal control which defines clear activation patterns for each muscle group involved.

These activation patterns are either set to fixed predefined intervals [7] or adjusted empirically [8–10]. The expected behaviour of the observed muscle group, as well as particular physiological limits (i.e. maximal leg extension and flexion) are taken into account when manually finding appropriate stimulation intervals. This initial fitting process is usually very time consuming and involves many iterations before autonomous cycling can be achieved. Such prolonged set-up procedures, can lead to increased muscular fatigue which then limits the cycling performance during the actual session—thus causing a less fulfilling user experience.

A method to overcome these obstacles, is to define stimulation patterns based on EMG recordings of healthy subject during voluntary cycling [11, 12], in an attempt to mimic the natural order of muscular activation. This assumes a similar force production between healthy and paralyzed muscles—which is not always the case.

Alternatively, it is possible to control stimulation by taking into account the actual movement (i.e. knee-extension, knee-flexion) during cycling. Previously, research groups have investigated the use of inertial measurement units (IMUs) to control stimulation in FES-Cycling, by assessing the thigh [13, 14] or knee angle [15, 16]. The motivation for this was to create a simpler definition of stimulation intervals, which automatically adapts for certain variations during cycling (e.g. changes in the seating position of the pilot). However, although the application of stimulation can be interpreted more intuitively, they also require a distinct definition of stimulating intervals.

To obtain optimal stimulation intervals, it is necessary to measure the instantaneous torque produced throughout a pedalling cycle [17–19]. The process can be achieved in two phases: a passive phase, which determines the torques while passively moving the legs and an active phase, which determines the torques during continuous stimulation. In both phases the rotational speed is kept constant by a motor that generates the movement. Subtracting the passive torque from the active torque, reveals a time-interval in which the stimulated muscle-group was actively contributing to the desired movement (i.e. a positive net-torque). This interval should be used for muscular activation. Due to the need for a dedicated motor control (constant angular velocity) and the requirement of measuring the instantaneous torque within the pedalling cycle, this approach was so far only implemented in stationary FES-Cycling systems.

The aim of the present work was to translate this stationary concept (i.e. fixed cadence) measurement into the domain of mobile FES-Cycling. We hypothesized that it is possible to use a commercially available crank power-meter to detect optimal stimulation intervals for both, crank-based and IMU-based stimulation control. Instead of a motor which rotates at a constant angular velocity as proposed in literature, the legs were moved manually by the investigator which makes the solution more widely applicable. Differences in angular velocity were normalized by an automatic detection algorithm, which analysed active and passive torques within a pedal turn. The functionality of the determined patterns was validated in one subject with complete SCI on a home trainer.

## Material and methods

### Subject

The proposed approach was tested on one male participant (40 years, 175 cm, 80 kg) with complete spinal cord injury (T4, ASI A). The conducted measurements were approved by the French national ethics committee (Comité de Protection des Personnes Sud-Est IV) as part of a wider scale study on the beneficial effects of FES-Cycling (CPP: 19.04.08.69902, ID-RCB: 2019-A00808-49). The participant gave his written informed consent prior to the testing. The participant performed a regular FES-Cycling training for > 12 months in preparation for the Cybathlon 2020, prior to the measurements [20].

## Setup

**Fig. 1:**
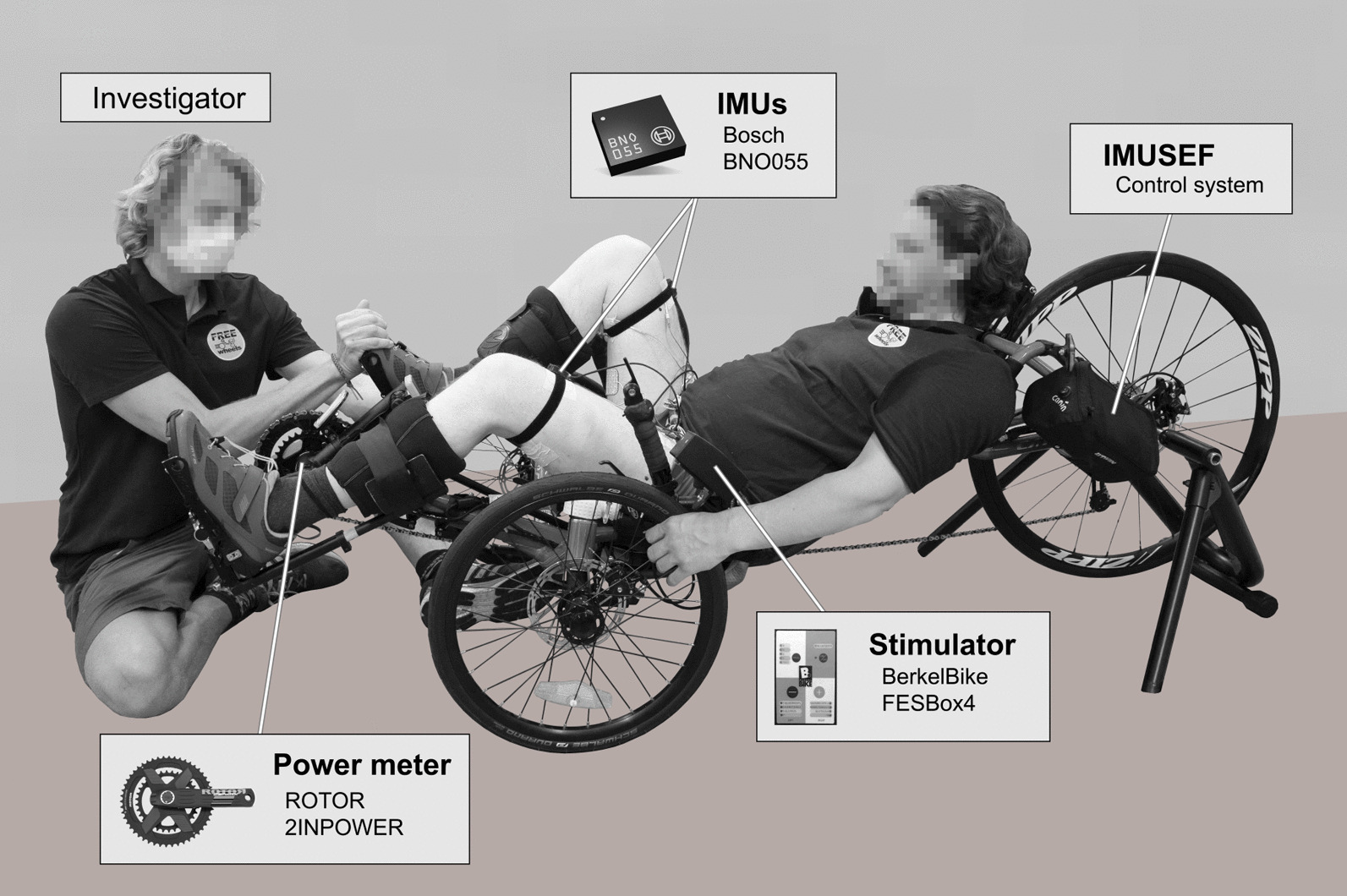
Measurement setup. A commercially available trike (CATrike 700) was adapted to allow for FES-Cycling in individuals with SCI. The crank-angle and bilateral torque were measured wirelessly by a power-meter (ROTOR 2INPOWER) at a sampling rate of 50 Hz. The thigh-angle was assessed bilaterally through 2 independent inertial measurement units (BNO055) at a sample-rate of 100 Hz. All data was processed by a Raspberry PI running the IMUSEF software (control system). (Image: © Inria / Photo H. Raguet)

A commercially available trike (CATrike 700, Big Cat HPV LLC, Orlando, FL, USA) was modified to allow for FES-Cycling in individuals with a complete spinal cord injury (see Fig. 1). The original crank set was replaced by a crank power-meter (2INPOWER, Rotor Bike Components, Madrid, Spain), without the need for further mechanical adaptations. Each leg was equipped with an inertial measurement unit (BNO055, Bosch GmbH, Gerlingen, Germany) located at the thigh. The IMUs processed (Kalman filter) inertial data at a sampling rate of 100 Hz, and were polled via its I2C-interface. Orthotic special pedals with calf support (Hase pedals version 1, Hase Bikes, Waltrop, Germany) were used to restrain movements of the ankle joint. The velocity was determined by a reed-switch which detected a magnet mounted on the rear-wheel of the trike. The trike was mounted onto a home trainer (In’Ride 100, B’TWIN, Decathlon, Villeneuve d'Ascq, France).

All data was handled by a Raspberry Pi (Model 3B, Raspberry Pi Foundation, United Kingdom) using a standard ANT + Dongle (ANTUSB-m, Garmin Ltd., Olathe, KS, USA), its I2C and GPIO interface. A software platform called IMUSEF (Inertial Measurement Unit pour Stimulation Électrique Fonctionnelle, CAMIN team, Inria, University of Montpellier, France), written in Python (Version 2.7) was responsible for data processing and controlling an electrical stimulator (FESBox 4, BerkelBike B.V., Netherlands) at a sample rate of 100 Hz. The software itself was controlled by a separate personal computer (MSI GS60 2PE Ghost Pro, Micro-Star International, Zhonghe District, Taiwan) which allowed for a graphical representation of the analysed data. For safety reasons an easily accessible emergency stop button was integrated in the handle bar.

### Crank angle, torque (power meter)

The crank-angle along with left and right torque was received wirelessly (ANT + communication) at a sample rate of 50 Hz (manufacturer specific “fast-mode”) from the power meter. For a wider academic access, we freely provide the source-code (Python 2.7) for communicating with the Rotor 2INPOWER [21]. A crank-angle of 0° corresponds to a vertical downwards facing position of the right crank-arm, with increasing values in clock-wise direction (seen from the right side). The power meter reveals a constant delay of 200 ms due to the wireless data-transmission.

### Cycling phase (IMU)

The cycling phase is a normalized representation of the thigh-angle (measured with IMUs) as described in [15]. For each cycle (i.e. full knee flexion–full knee extension–full knee flexion), the minimal and maximal thigh angle values are used to calculate a relative position within the cycle. Therefore, knee-extension is described by values from 0 to 50%, while knee-flexion correlates to values from 50 to 100%. Please note that the signals obtained by the IMUs are delayed by a constant of 40 ms due to a Kalman filter implemented in the IMU module (i.e. Bosch BNO055 sensor).

### Optimal interval detection algorithm (OIDA)

The optimal interval detection algorithm (OIDA) interprets torque measurements in combination with a correlated input signal. For each muscle (or muscle group) of interest, passive (without stimulation) and active (with continuous stimulation) torques were recorded during consecutive pedalling cycles with the crank power-meter. By calculating the net-torque (i.e. subtracting passive torque from active torque), it is possible to determine the positions at which the stimulated muscle is actively contributing to the cycling motion. If the net-torque is negative, the muscle is resisting the cycling motion, thus one should not stimulate at these positions. The optimal interval for delivering electrical stimulation is therefore the range of positions where the net-torque is positive. In contrast to previous works [17–19] detecting automatic stimulation intervals on motorised ergo-cycles, our approach is not relying on a constant angular velocity provided by a motor and allows to process alternative control signals (i.e. cycling phase measured via IMUs).

**Fig. 2:**
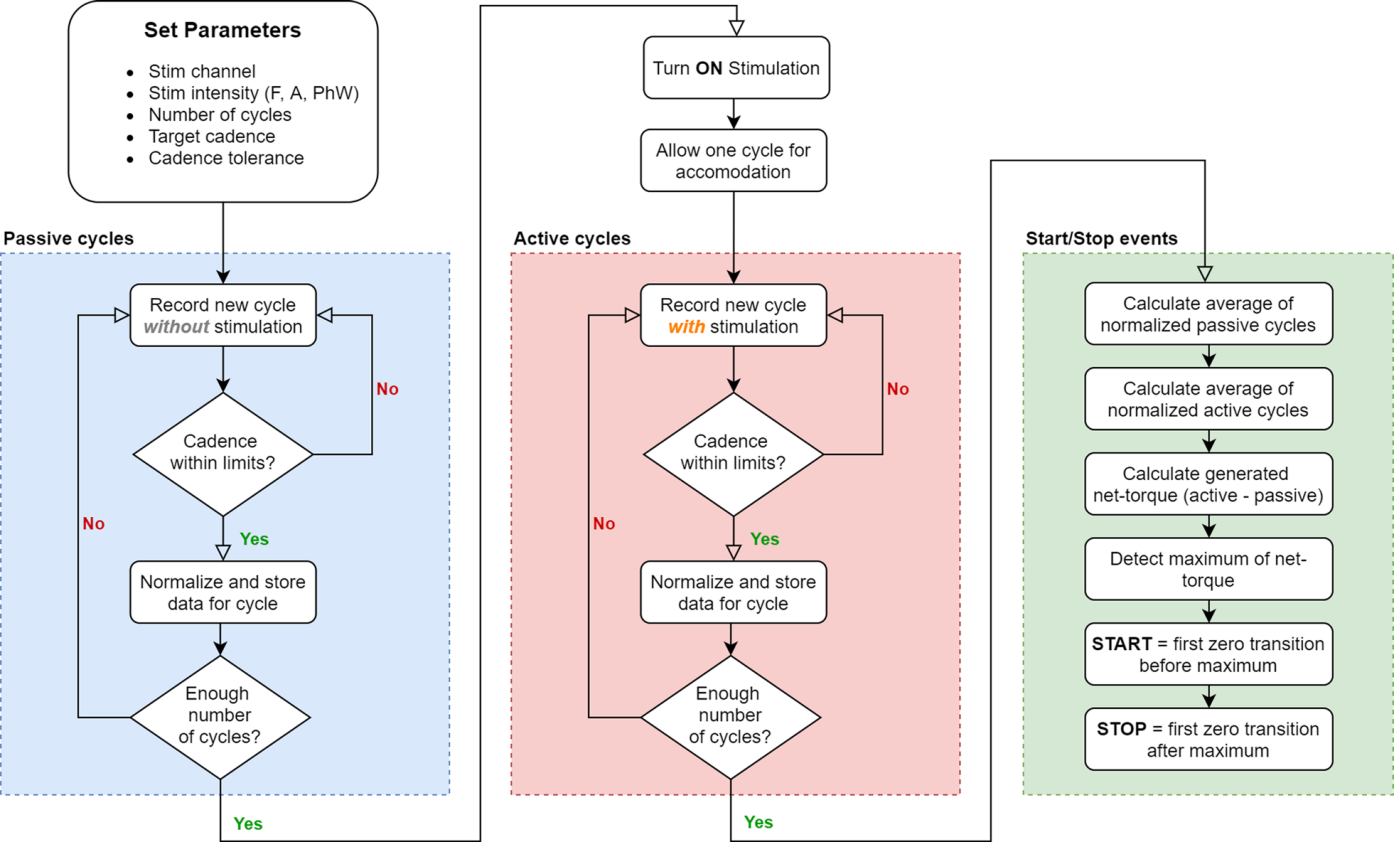
Flowchart of the OIDA. Schematic representation of the optimal interval detection algorithm. After setting the parameters, a predefined number of passive (without stimulation) and active (with continuous stimulation) cycles are recorded. The net-torque (i.e. active torque minus passive torque) is used to determine an optimal interval (i.e. start and stop value of the crank-angle) for applying stimulation

The algorithm, as shown in Fig. 2, was integrated into our existing FES-Cycling platform (IMUSEF) as a software module (Python 2.7). As the main software itself, OIDA operates at a sampling frequency of 100 samples / s. Due to the different delays of the input signals obtained from the power-meter and the IMU sensors, it was necessary to align the recorded data prior to processing. Thus, the cycling phase data was shifted by 40 ms, while the data for crank angle and torque were shifted by 200 ms. The torque distribution of a muscle was recorded throughout a pedalling cycle, while the contralateral leg was passively moved by the investigator.

The implementation of the OIDA allowed to select: stimulation channel, stimulation parameters, number of cycles, target cadence and cadence tolerance. The stimulation channel defined the targeted muscle or muscle group. The stimulation parameters (i.e. frequency, amplitude and phasewidth) defined the strength of the contraction. Through the number of cycles it is possible to obtain a result based on the average of multiple cycles. A valid cycle is only accepted if the turning motion is within a specified cadence range (target cadence ± cadence tolerance).

After recording torque data for the specified number of passive cycles, stimulation was switched ON and the same number of active cycles was recorded during continuous electrical stimulation. Due to an expectable sudden movement at the onset of stimulation, the first active cycle is rejected from analysis. To compensate for different cycle durations, each full cycle was interpolated to match a template vector (i.e. crank-angle: 0–360° in 1° steps; cycling-phase: 0–100% in 1% steps). The mean torque of all normalized passive cycles and the mean torque of all active cycles were used to calculate the net-torque (i.e. active contribution of the stimulated muscle). OIDA automatically detected the optimal start and stop position, by searching the zero-transitions closest to the maximal net-torque.

### OIDA measurements

**Fig. 3:**
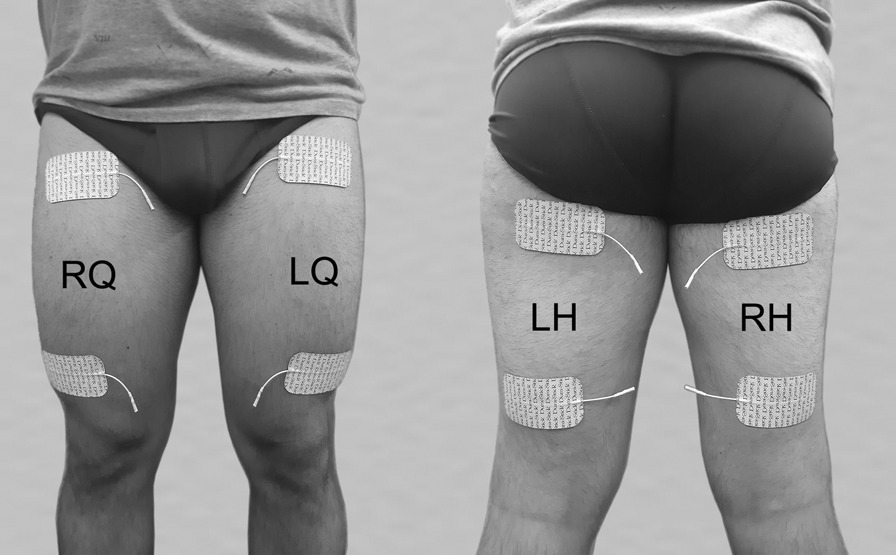
Electrode positioning. Pairs of stimulating hydrogel electrodes were placed on the quadriceps (LQ, RQ) and hamstring (LH, RH) muscles

The pilot was comfortably seated onto the trike with his legs safely secured by special orthotic pedals with calf support. Pairs of stimulating hydrogel-electrodes (Durastick 50 × 90 mm, DJO International, Guildford Surrey, UK) were placed bilaterally over the quadriceps and hamstring muscles as demonstrated in Fig. 3. This electrode placement was chosen as it is widely used in clinical practice for muscle strengthening and FES-Cycling.

The current-controlled stimulator was set to deliver biphasic rectangular charge-balanced pulses with a phasewidth of 400 µs and a stimulation amplitude of 72 mA at a frequency of 40 Hz, aiming to induce a forceful muscular contraction. The OIDA controlled the stimulation for each targeted muscle. For safety reasons it was possible to stop stimulation at any time via an emergency-stop button on the handle bar of the trike.

Each muscle (i.e. left quadriceps LQ, right quadriceps RQ, left hamstrings LH and right hamstrings RH) was tested separately by passively moving the contralateral leg. The algorithm recorded 3 passive and 3 active (with stimulation) cycles at a cadence of 5 RPM with a tolerance of 50% for each muscle. If the examiner was moving the leg too fast (> 7.5 RPM) or too slow (< 2.5 RPM) the cycle was rejected. After a successful measurement, the recorded data was graphically presented along with the determined stimulation intervals.

### Cadence dependent delay compensation

The stimulation pattern obtained by the OIDA describes an interval of crank-angles or cycling-phase-values, at which the corresponding muscles should be active in order to produce a positive net-torque. As these intervals were obtained at a very low cadence, it is necessary to compensate potential delays to allow for smooth cycling at higher cadences.

Sensor delays (Delay_Sensor_) amounted to: (a) 200 ms for the crank power-meter (i.e. crank-angle) due to the wireless data transmission or (b) 40 ms for the signals obtained by the IMUs (i.e. cycling-phase) due to the Kalman filtering implemented in the BNO055 modules. Furthermore, the muscle itself also requires a certain amount of time (Delay_Muscle_) to be active (between 100 and 300 ms [22]). In our application we empirically estimated a muscular delay of 200 ms for our pilot.

To guarantee for an active muscle at the correct positions, it is important to start the stimulation earlier than defined by the stimulation pattern. The required shift is depending on the duration of the current cycle (Duration_Cycle_) and can be calculated using Eq. 1. When starting the stimulation, it is necessary to compensate for the muscular delay and sensor delay. For stopping the stimulation, it is sufficient to compensate for the sensor delay only. The corresponding shift can be calculated using Eq. 2.

1 $${Shift}_{START}=\frac{\left(Dela{y}_{Muscle}+Dela{y}_{Sensor}\right)}{Duratio{n}_{Cycle}}*360^\circ ,$$

2 $${Shift}_{STOP}=\frac{Dela{y}_{sensor}}{Duratio{n}_{Cycle}}*360^\circ$$

### Validation of stimulation patterns

The stimulation patterns (i.e. start and stop values for each muscle group) obtained by the OIDA were validated during a 5 min FES-Cycling test without load on a home trainer. The aim of this test was to determine whether the stimulation patterns obtained by the OIDA allowed for stable and autonomous cycling—i.e. without interventions of the pilot or examiner (e.g. manually pushing the legs to avoid dead points).

After manually initiating the cycling motion by the examiner, IMUSEF (control software for FES-Cycling) delivered delay compensated stimulation according to the previously determined pattern. The stimulation amplitude was gradually increased by the pilot to allow for autonomous cycling at a targeted cadence of between 50 and 60 RPM. To counteract fatigue, the pilot was instructed to adjust the stimulation amplitude towards a maximum of 120 mA. Crank-angle; left and right thigh-angle; left and right torque; power per cycle; cadence per cycle; speed and stimulation amplitude were recorded throughout the entire measurement.

## Results

### Optimal stimulation intervals

**Fig. 4:**
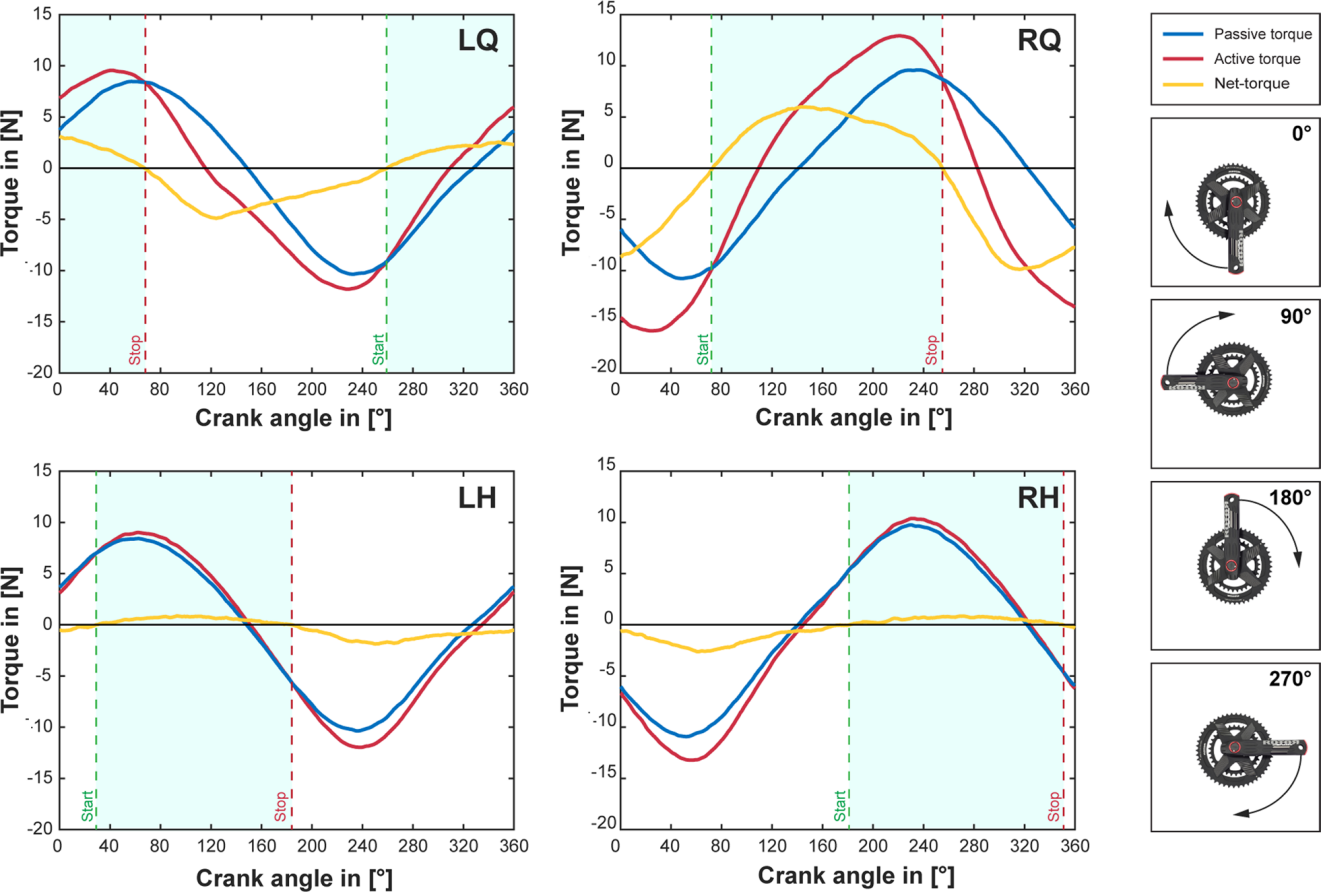
Results of the OIDA measurements for crank angle. Illustration of the results of the optimal interval detection algorithm. Data is shown for the muscle groups: left quadriceps (LQ), right quadriceps (RQ), left hamstrings (LH) and right hamstrings (RH). The shown torque traces represent the average of 3 cycles. Blue traces correspond to passive cycles (without stimulation), red traces to active cycles (with stimulation) and orange traces to the generated net-torque (active minus passive cycles). Start and stop values define the interval in which the measured muscle group should be active (light blue shading). Please note: A crank-angle of 0° corresponds to a vertical downwards facing position of the right crank-arm, with increasing values in clock-wise direction (seen from the right side)

**Fig. 5:**
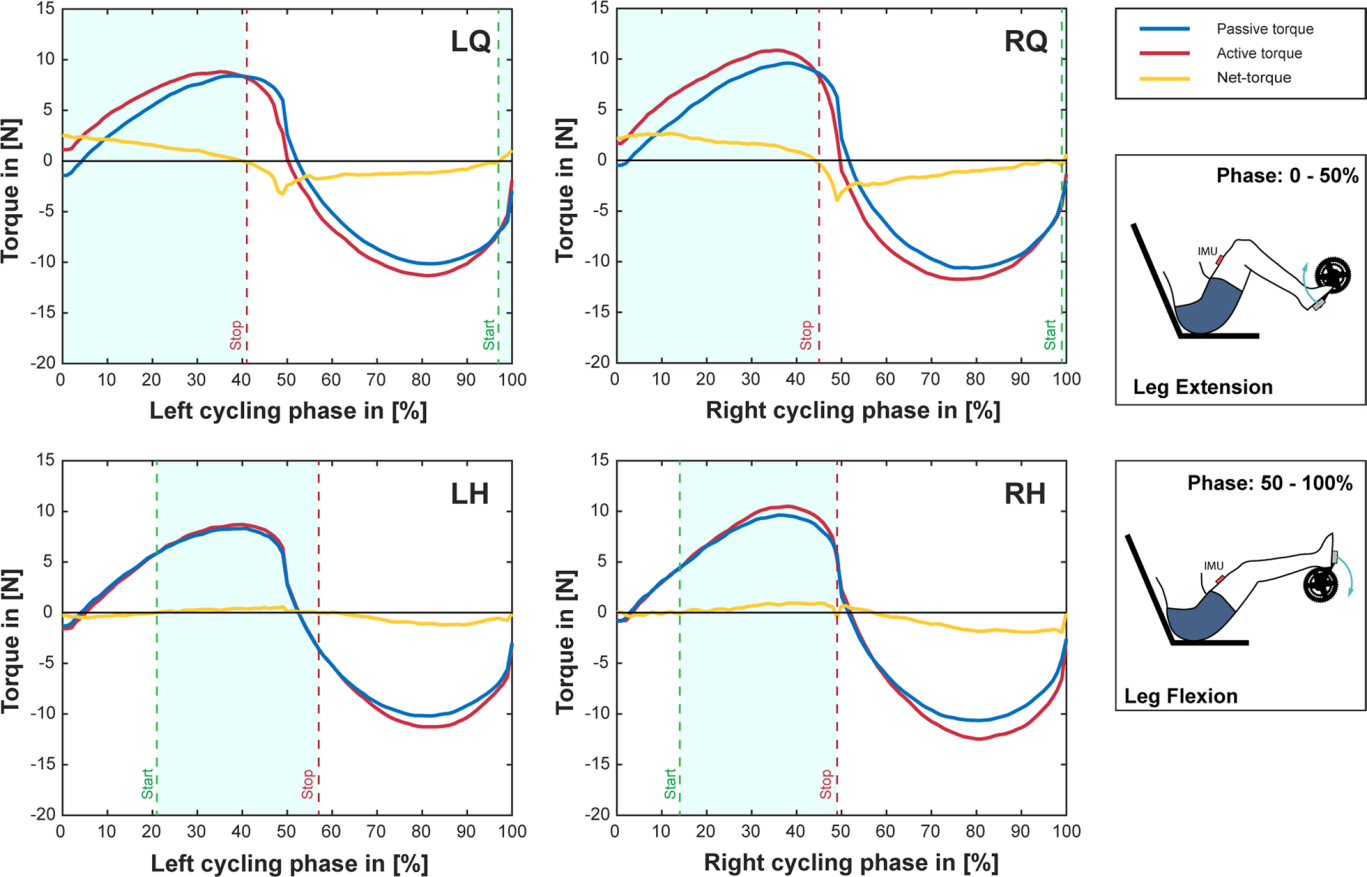
Results of the OIDA measurements for cycling phase (IMU based). Illustration of the results of the optimal interval detection algorithm. Data is shown for the muscle groups: left quadriceps (LQ), right quadriceps (RQ), left hamstrings (LH) and right hamstrings (RH). The shown torque traces represent the average of 3 cycles. Blue traces correspond to passive cycles (without stimulation), red traces to active cycles (with stimulation) and orange traces to the generated net-torque (active minus passive cycles). Start and stop values define the interval in which the measured muscle group should be active (light blue shading). Please note: Cycling phases are calculated individually for left and right side (independent IMU´s). The first 50% of the cycling phase represent leg-extension, while the last 50% indicate leg-flexion

A custom-developed algorithm for detection of optimal stimulation intervals was used to determine a stimulation pattern for FES-Cycling involving the quadriceps and hamstrings muscles. Figures 4 and 5 graphically present the results for each investigated muscle group and indicate the optimal intervals for stimulation between the start and stop markers, respectively for crank-angle or cycling-phase based stimulation. Data was obtained from 3 passive (without stimulation) and 3 active (with continuous stimulation) cycles and averaged to reduce potential variation. The corresponding values of the stimulation patterns can be found in Table 1. Please note that the OIDA measurements for the different control signals (i.e. crank angle and cycling phase) were performed at different time points throughout a single measurement day, thus causing differences in the peak values of the measured torques.

**Table 1:** Stimulation patterns obtained by the OIDA

	Crank angle		Cycling phase (IMU based)	
	Start [°]	Stop [°]	Start [%]	Stop [%]
LQ	259	68	97	41
RQ	72	255	99	45
LH	29	184	21	57
RH	181	351	14	49

### Validation of the stability of the stimulation pattern

**Fig. 6:**
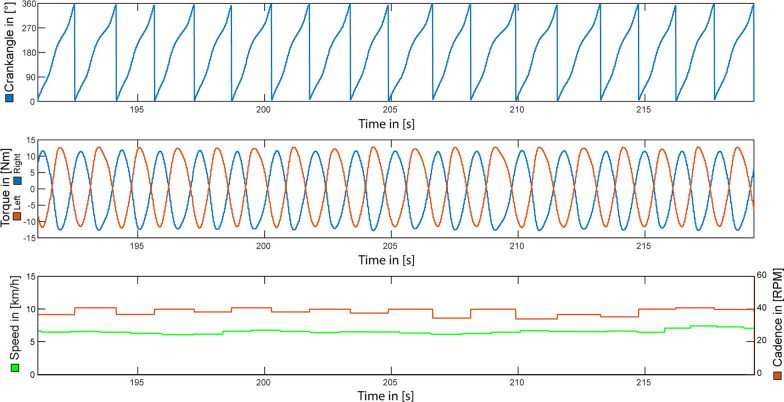
FES-Cycling controlled via crank angle. Representative data for approximately 30 s of FES-Cycling on a home trainer without resistance. Stimulation pattern was determined automatically by the OIDA based on the crank angle

**Fig. 7:**
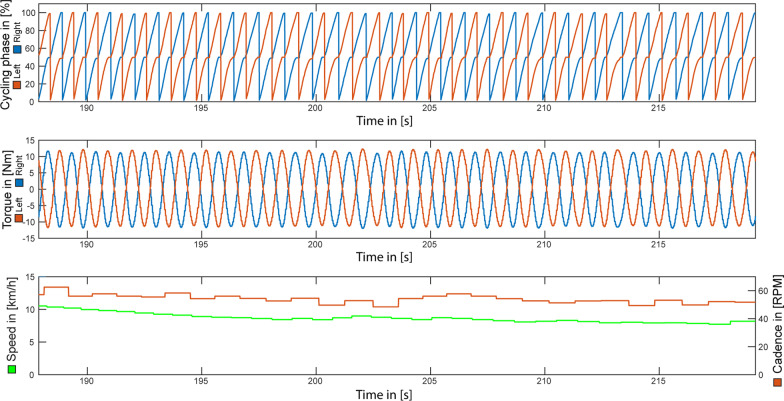
FES-Cycling controlled via cycling phase (normalized thigh-angle). Representative data for approximately 30 s of FES-Cycling on a home trainer without resistance. Stimulation pattern was determined automatically by the OIDA based on the cycling phase. Please note: The cycling phase is a normalized representation of the thigh angle, which is measured via IMUs individually for left and right side

Both stimulation patterns determined by the OIDA allowed for stable cycling without further adaptation. The movement was initiated manually by the examiner while the subject controlled the stimulation intensity until the muscular contraction was sufficient to maintain the cyclic motion. The cycling motion was stable for the entire duration of the 5 min cycling test—i.e. no manual intervention was required by neither the pilot nor the examiner. For crank-angle based stimulation, autonomous cycling started with an initial stimulation intensity of 76 mA and was increased to the maximum of 120 mA within the first 91 s. For cycling-phase based stimulation, autonomous cycling was achieved at a stimulation amplitude of 65 mA, and increased to the maximum of 120 mA within 171 s. Figures 6 and 7 shows exemplary data measured during autonomous cycling without load, respectively for crank-angle and cycling-phase based stimulation. A summary of the measured results for the different control approaches is provided in Table 2.

**Table 2:** FES-Cycling with different control modalities

Control	Duration	Distance	Speed	Cadence	Power			
			AVG	MAX	AVG	MAX	AVG	MAX
	[mm:ss]	[m]	[km/h]	[km/h]	[RPM]	[RPM]	[W]	[W]
Crank Angle (Rotor)	05:03	654.9	7.8	14.0	45. 7	75.0	1.58	4.37
Cycling Phase (normalized thigh-angle, IMU)	05:07	734.7	8.6	14.6	52.8	71.9	2.03	3.73

Summarized results for a 5 min autonomous FES-Cycling test on a home trainer without load, comparing the control modalities crank angle and cycling phase. The stimulation patterns for both input signals were determined automatically by the OIDA

## Discussion

Within this article we presented an algorithmic approach for automatically detecting stimulation intervals by evaluating the torque measured by a commercially available crank power-meter installed on an instrumented trike. We described the general methodology to determine stimulation patterns using the crank-angle or a normalized thigh-angle (cycling phase, measured via IMUs) as input signal. FES-Cycling was possible without further adaptation of the patterns, after only a short (~ 15 min) initial fitting phase to determine the stimulation pattern. In comparison to the empirical approach, in which the stimulation intervals are to be adapted iteratively for each individual muscle group, the OIDA was perceived as a useful tool which helped to improve the user-experience for both the pilot and the examiner. Since the obtained stimulation patterns are based on the actively generated torque, the OIDA could provide valuable guidance leading to reduced durations and increased compliance during the fitting procedure.

Our approach is not relying on a constant angular velocity as the algorithm is mapping the recorded torque-values onto a normalized data vector. Nevertheless, the investigator moves the pedals as constantly as possible to stay within a predefined tolerance – additionally taking care to avoid abrupt motions or stops. In contrast to motor-controlled systems [17–19], our approach has the advantage of a reduced system complexity, lower costs and weight due to not requiring a motor. However, it is important to acknowledge that certain variations in speed might affect the recorded torques. It is to expect that during stimulation the highest angular velocity within the cycle can be found around the positive peak-torque—due to the additional contributing action of the activated muscles. Thus, at these moments, a slightly lower net-peak-torque can be expected.

On the other hand, it can be argued that at the zero-crossings of the net-torque (i.e. most vulnerable positions, defining the optimal stimulation interval) no additional contributing or resisting forces are developed by the stimulated muscles. At these positions the examiner is only confronted with the passive forces, generated by the weight of the leg. To avoid additional muscular fatigue, we did not determine stimulation patterns empirically.

The crank angle intervals of positive net-torque determined by the OIDA algorithm (see Table 2) are comparable to previously reported values for LQ (285–105°), RQ (105–255°), LH (45–190°) and RH (200 to 355°) obtained with a stationary system [19]. Note that these intervals were graphically estimated based on their Fig. 2 [19]. Observable deviations are likely to be associated with differences in pilot position (i.e. sitting upright versus recumbent position), range of motion of the knee and the geometry of the different devices (i.e. ergo-cycle versus recumbent trike).

For the IMU-based cycling phase, instantaneous torque data throughout a pedalling cycle has not been reported yet. Nevertheless, Wiesener and Schauer suggested stimulation intervals for the quadriceps (0–40%) and hamstring (50–100%) muscles to be used for the cycling phase [15]. These intervals are based on simulations using a biomechanical model [23]. While our automatically determined cycling phase intervals (see Table 2) fit well for the quadriceps muscles, we do see distinct differences for the hamstrings. A possible explanation could be that the used model assumed a more idealised function of the hamstrings as knee-flexor, than actually achievable through surface electrodes.

A potential limitation of the presented study could be seen in the algorithm being validated in only a single subject. However, as the algorithm determining the stimulation intervals is based on the individually measured torques, it is reasonable to assume that the OIDA can be used to determine stimulation patterns customized also for other users. The general idea of the algorithm already has been demonstrated by other research groups using a constant angular velocity during stationary cycling [17–19]. Our approach therefore extends the concept to the domain of mobile FES-Cycling, additionally allowing for alternative control signals (e.g. cycling phase measured IMUs) as input. We further would like to emphasise that, the intervals obtained by the OIDA are to be considered optimal with respect to the generated active torque at the particular time point of the measurement.

In FES-Cycling, the quadriceps muscles in their function as knee-extensors generally account for the majority of the generated power. Through accurate switching of the stimulation it is possible to cycle exclusively using the quadriceps muscles alone [24]. The inclusion of additional muscle-groups to gain further improvements is not a trivial task. When done empirically, often an idealized function is assumed without actual verification. The hamstring muscles are another popular example to be used additionally for FES-Cycling. Unfortunately, the hamstrings reveal a much less predictable behaviour than the quadriceps muscles [24]. While from a physiological point they are considered as one of the main contributors to knee-flexion [25] – it is particularly difficult to achieve this isolated function when using electrical stimulation with surface electrodes. Slight deviations in the electrode positioning might cause even undesired effects counteracting the intended motion [24]. Correctly timed delivery of stimulation pulses is therefore crucial for successful cycling. As can be seen in Figs. 4 and 5, the negative (resisting) net-torque is generally greater than the positive (contributing) net-torque. As during resisting phases the muscle is acting eccentrically, it is able to generate higher torques than during contributing (concentric) phases [26]. Another interesting phenomenon, which can also be observed in Figs. 4 and 5, is a noticeable overlap in stimulation intervals between quadriceps and hamstring muscles of the same side. Such overlaps have also been previously reported in studies with manual [27] and automatic [18, 19] determination of stimulation intervals; and even with implanted stimulation systems [28]. While a certain degree of co-contraction can be expected to be beneficial for knee stability, it might also indicate a sub-optimal electrode placement. A poorly timed stimulation pattern will therefore cause additional fatigue or even abruptly terminate the cycling motion.

With the proposed algorithm, it is possible to quantitatively assess the contribution of each stimulated muscle group and thus verify whether a chosen electrode configuration is in fact producing the desired output. In future research, the OIDA could be used to systematically include additional muscles, such as the gluteus maximum muscles, or to investigate the influence of fatigue on the instantaneous torque production throughout a pedalling cycle. In this study we focussed on the description and a functional assessment of the algorithm itself.

The stability (i.e. ability for uninterrupted autonomous cycling) of the stimulation patterns was validated during a 5 min cycling test on a home trainer. The performance data presented in Table 2, is to be interpreted as additional support for the functionality of the automatically determined stimulation patterns. A quantitative comparison of the cycling performance of the different control modalities (i.e. crank angle versus cycling phase) is not possible, as the measured data was obtained at different time points throughout a single measurement day (i.e. different initial states of muscular fatigue).

All tests were performed without resistance from the home trainer. Load-less cycling introduces a particular challenge, as even slightly miss-tuned stimulation patterns will have a great influence on the pedalling smoothness. Nevertheless, we acknowledge the fact that over-ground cycling also should be assessed. Due to time constraints, we only were able to demonstrate over-ground cycling with the IMU-based (i.e. cycling phase) pattern (see video in Additional file 1). With this automatically determined stimulation pattern, our pilot was able to cycle on flat ground for 201.6 m in 03:58 min. After a manual start from a standing position (using push-buttons), he cycled with an average speed of 3.05 km/h (max. 6.49 km/h), at an average cadence of 25.6 RPM (max. 56.1 RPM), generating an average power of 9.3 W (max. 33.1 W).

Finally, we would like to highlight the power-meter (Rotor 2INPOWER) because it is currently the only commercially available power-meter offering a ‘fast-mode’ which allows capturing a variety of data at a comparably high sample-rate of 50 Hz. This is in contrast to other high-end power-meters like the Garmin Vector 3 or PowerTap P1, which only broadcast their information at a maximum rate of ~ 4 Hz. While this sampling frequency is in accordance with the specifications for the ANT + power profile and certainly sufficient to assess sportive performance—it is not possible to analyse the torque-generation within a pedalling cycle. Thus, the Rotor 2INPOWER fills a gap which can help to improve quantitative assessment of power within a pedalling cycle. Although in this study we only used information about the crank-angle and torque, we would like to point out that also other parameters (e.g. power, tangential force, cadence, pedal-smoothness, pedal-balance…) are available, which might be of interest in other studies. In order to facilitate its usage, we freely provide the source code (Python) for communication (ANT +) with the power-meter—including an installation guide online available at [21].

## Conclusions

Within this work we demonstrated an algorithmic approach to determine optimal stimulation intervals for FES-Cycling, taking into account the instantaneous torque, generated through electrical stimulation. In contrast to other studies, our approach does not require a constant angular velocity during recording. Like this, it can be used in mobile FES-Cycling solutions, with an examiner passively moving the legs of the subject during a fitting session. As the measurements were performed on a commercially available power-meter, our algorithm could be easily implemented into other systems, allowing for decreased setup times and more efficient cycling in comparison to a manual setup. In this study, we demonstrate the feasibility of automatic stimulation pattern detection, using the crank-angle and a normalized thigh-angle (measured via IMU´s) as an input signal. Both stimulation patterns allowed for autonomous FES-Cycling in a pilot with complete spinal cord injury, without the need for further adaptations.

## Supplementary Information

**Additional file 1.** Video demonstration of IMU-based overground FES-Cycling.

## Data Availability

All required data and material has been included in the manuscript. High quality copies of the figures are submitted in separate files enclosed to the manuscript. The source code (Python 2.7) for communicating with the power meter 2INPOWER from Rotor as well as an installation guide is made available online at https://gitlab.inria.fr/camin-soft/ant4rotor. The software package allows for connection and configuration of the power meter in order to receive data at a sample rate of 50 Hz using its “fast-mode”.

## Abbreviations

A: Stimulation Amplitude
EMG: Electromyography
F: Frequency
FES: Functional electrical stimulation
IMU: Inertial measurement unit
IMUSEF: Inertial Measurement Unit pour Stimulation Électrique Fonctionnelle
LH: Left hamstrings
LQ: Left quadriceps
OIDA: Optimal interval detection algorithm
PhW: Phasewidth
RH: Right Hamstrings
RPM: Rotations per minute
RQ: Right quadriceps
SCI: Spinal cord injury
SD: Standard deviation
